# Ion-Trap Mass Spectrometric Analysis of Bisphenol A Interactions With Titanium Dioxide Nanoparticles and Milk Proteins

**DOI:** 10.3390/molecules25030708

**Published:** 2020-02-06

**Authors:** Edward P.C. Lai, Hendrik Kersten, Thorsten Benter

**Affiliations:** 1 Ottawa-Carleton Chemistry Institute, Department of Chemistry, Carleton University, Ottawa, ON K1S 5B6, Canada; 2 Institute for Pure and Applied Mass Spectrometry, Physical and Theoretical Chemistry, Bergische Universität Wuppertal, Gaussstr. 20, 42119 Wuppertal, Germany; hkersten@uni-wuppertal.de (H.K.); tbenter@uni-wuppertal.de (T.B.)

**Keywords:** bisphenol A, ion-trap mass spectrometry, milk whey proteins, sodium formate, titanium dioxide nanoparticles

## Abstract

Quantitative analysis of endocrine-disrupting molecules such as bisphenol A (BPA) in freshwater to determine their widespread occurrence in environmental resources has been challenged by various adsorption and desorption processes. In this work, ion trap mass spectrometry (ITMS) analysis of BPA was aimed at studying its molecular interactions with titanium dioxide (TiO_2_) nanoparticles and milk whey proteins. Addition of sodium formate prevented TiO_2_ nanoparticles from sedimentation while enhancing the electrospray ionization (ESI) efficiency to produce an abundance of [BPA + Na]^+^ ions at *m*/*z* 251.0. More importantly, the ESI-ITMS instrument could operate properly during a direct infusion of nanoparticles up to 500 μg/mL without clogging the intake capillary. Milk protein adsorption of BPA could decrease the [BPA + Na]^+^ peak intensity significantly unless the proteins were partially removed by curdling to produce whey, which allowed BPA desorption during ESI for quantitative analysis by ITMS.

## 1. Introduction

The widespread occurrence of bisphenol A (BPA) in freshwater resources can cause serious public health problems even at low exposure levels [1]. BPA is an important chemical that is still widely used for the industrial manufacture of diverse consumer products despite its known toxicity [2]. Regarding human exposure to BPA, food intake can be considered the most serious, not only because it potentially reaches more people in different age groups, but also because it inadvertently occurs over long time periods [3]. Recently, researchers in Italy studied the occurrence of BPA in milk; their results suggested that new monitoring plans should be applied at each stage of the milk chain [4]. Vegetable oil, canned fish, and canned meat may have BPA concentrations as high as 20–30 ng/g [5]. The compound mimics estrogens in the human body, leading to adverse health effects such as polycystic ovary syndrome and thyroid cancer, which are common among women [6,7,8]. Exposure of humans to BPA has also been associated with increased weight and obesity [9]. Biochemically, BPA upregulates the expression of factors involved in the inflammatory pathway, thus helping in the progression of cancer [10]. It causes the lysosomal degradation of hypoxia-inducible factor-1α by heat-shock cognate protein [11]. It also induces DNA damage and activation of signaling pathways that initiate tumorigenic changes in human cells [12]. In the European Union, BPA is severely restricted by law due to its endocrine-disrupting properties. Commission regulation 2018/213 amended the previous UE regulation 10/2011 on the level of BPA in plastic food contact materials, reducing the specific migration limit from 0.6 to 0.05 mg BPA/kg [13]. Electrochemical sensors and liquid chromatography have increasingly been used in the last several years for the quantification of BPA in water and milk analysis [14,15]. High-performance liquid chromatography-tandem mass spectrometry methods can determine BPA, its major metabolite BPA-glucuronide, and eleven BPA analogues in human urine and blood serum [16,17]. Other analytical methods for the detection of BPA at trace levels in solid environmental matrices have recently been reviewed [18].

There is mounting evidence that adsorption of BPA onto various solid particles can jeopardize the accuracy of quantitative water analysis [19]. More importantly, engineered nanomaterials such as titanium dioxide (TiO_2_) and mixed metal oxides may possess binding properties towards BPA [20,21,22]. On the other hand, trace BPA in wastewater samples could be preconcentrated with magnetic iron oxide (Fe_3_O_4_)-graphene oxide nanocomposite or magnetite nanoparticles as the adsorbent [23,24]. Sorption of BPA by methacrylate-functionalized Fe_3_O_4_ magnetic nanoparticles was fast and efficient (Q_max_ = 600 mg/g), yielding a removal efficiency of ~98% in 40 min of immersion [25]. Activated carbon, bioderived porous carbon, biomass-derived biochars, CdS-hydrogel, β-cyclodextrin porous polymer, modified goethite nanorods, and graphene aerogel have all been evaluated for the removal of BPA from water [26,27,28,29,30,31,32,33,34]. Generally, the adsorption process best fits in a pseudo-second-order rate equation, and the adsorption equilibrium data fits the Langmuir monolayer or Freundlich adsorption model. Since BPA adsorption is a spontaneous exothermic process, the adsorption capacity decreases with increasing temperature. The largest adsorption of BPA by poly-2-vinylpyridine-functionalized magnetic nanoparticles was attained at pH 5.0, probably due to hydrogen-bonding interactions [35]. A pH value of 3–8 is conducive to the adsorption of BPA on mesoporous carbon, as the principal adsorption mechanism is H-bonding [36]. Due to the high K_ow_ value of BPA, the synergy of hydrogen bond-forming alkaline imidazole groups and hydrophobic hexadecyl groups benefits BPA removal from aqueous solutions using silica (SiO_2_) in the pH range of 4.0–9.0 [37]. Extraordinary cation-π electron interaction also contributes substantially to BPA adsolubilization on CTAB-modified graphite [38].

Desorption of BPA from solid nanoparticles during water sample pretreatment is regarded as the panacea for quantitative analysis in all serious environmental studies. It has been reported that either ethanol solution (75%) or ammonium hydroxide solution (0.5 M) is capable of desorbing BPA off carbon adsorbents [36]. Indeed, the BPA recovery rate increased with increasing solvent polarity, indicating a stronger interaction with BPA when the number of available polar sites in the solvent molecule became greater [39]. At pH ≤ 8, most of the BPA (pK_a_ 9.7) present is neutral, resulting in decreased binding. Hence, a pH between 5 and 8 is suitable for desorption. The objective of this study was to investigate the fundamental interactions of BPA with TiO_2_ nanoparticles in the process of developing an accurate method for the determination of BPA in complex heterogeneous sample matrices. TiO_2_ (as opposed to other nanoparticles) was chosen in the present work, because it is commonly used in water purification to mitigate organic contaminants, to remove humic acid, and to photodegrade pesticides [40,41,42,43]. TiO_2_ is also commonly applied as a nondairy whitener to enhance the brightness of food products (including cake icing/frosting, cheese, ice cream, skim/soy milk, and yogurt) and as a white pigment in personal care products (such as toothpaste) [44,45].

Ion trap mass spectrometry (ITMS), used alone without coupling to liquid chromatography, was employed in the present study to investigate BPA adsorption onto both TiO_2_ nanoparticles and milk proteins. Over the past decade, ITMS has become a powerful research tool for the characterization of molecular structures in physical–analytical chemistry. Numerous reports can be found in the literature that illustrate the use of high-resolution tandem mass spectrometry (MS/MS) for the characterization of proteins [46,47,48,49], identification of peptide [50,51,52], untargeted screening of lipids [53], investigation of rhamnogalacturonan II pectin [54], and quantification of transfucosylation products for manufacturing biomimetic human milk oligosaccharides [55]. Other investigations varied from the synthesis of diarylethene fluorescence probes with a methylquinoline moiety [56], analysis of intermediates produced in the sulfamethoxazole photodegradation process [57], identification of isopropylphenol oxidation products [58], analysis of carotenoids in citrus [59], and chemical structure confirmation of phenolic compounds [60] to a wide range of bioanalytical applications using electrospray ionization (ESI) [61,62,63,64,65,66,67]. This literature review emphasizes both the versatility of ESI-ITMS as an analytical tool and the novelty of our BPA adsorption/desorption study uniquely with TiO_2_ nanoparticles or milk proteins. Moreover, BPA molecules have a propensity for forming adduct ions with alkali metal ions to facilitate ionization in mass spectrometry (MS). As the compound gives a low abundance of measurable fragments, it would be worthwhile to investigate using the sodium adduct ion for accurate quantitative MS analysis after adding a small excess of sodium formate (or acetate) to the sample solution [68,69]. The general purpose of adding sodium formate (or acetate) was for the enhancement of MS signal intensity. Sodium formate can also be used to calibrate mass spectrometers across the mass-to-charge ratio (*m*/*z*) range of 50–1200 [70].

## 2. Results and Discussion

### 2.1. Electrospray Ionization Ion Trap Mass Spectrometry

**Figure 1:**
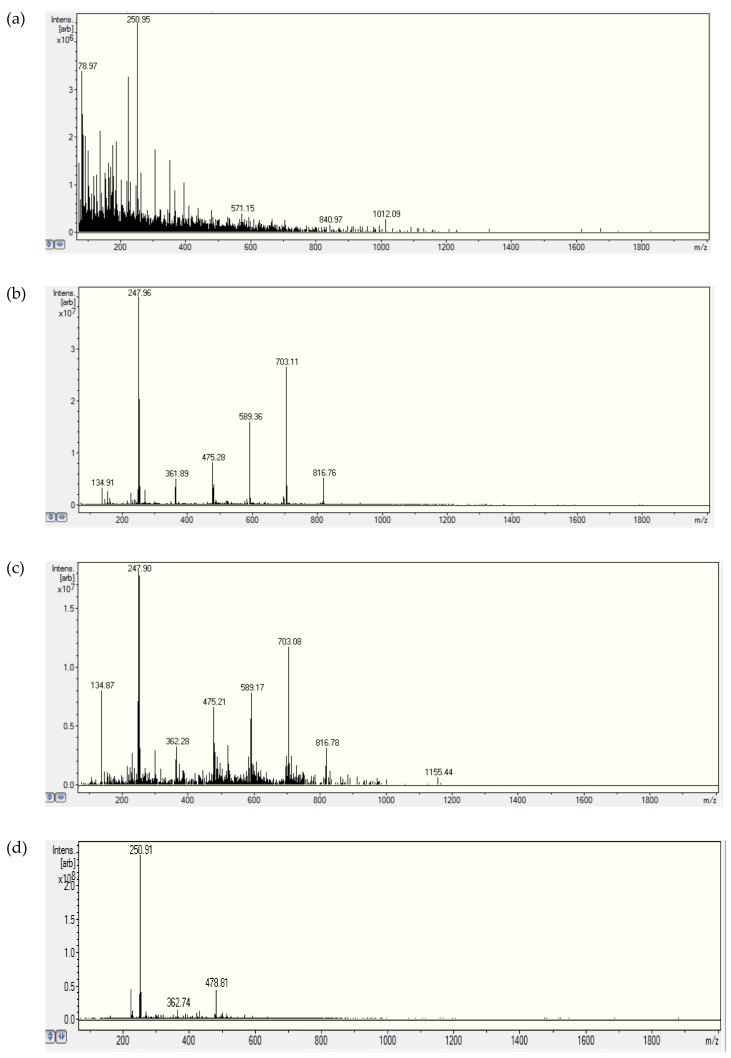
Electrospray ionization-ion trap mass spectronomy (ESI-ITMS) analyses: (**a**) Millipore water, (**b**) bisphenol A (BPA) (100 μg/mL), (**c**) BPA (100 μg/mL) + TiO_2_ nanopowder (414 μg/mL), (**d**) BPA (67 μg/mL) + TiO_2_ nanopowder (276 μg/mL) + sodium formate (50 μg/mL).

Ion trapping (IT) is a dynamic technique that renders the mass spectrometer (MS) a very sensitive detector of molecular ion and fragments, resulting in the electrospray ionization (ESI) source. Although an ion trap mass spectrometer was used in this study, all the quantitative analyses was performed in a single-ion monitoring mode, and samples were directly infused into the mass spectrometer without precedent chromatography. Fortunately, no significant spectral interference was observed from blank sample matrices. During the present investigation of the fundamental interaction between BPA and TiO_2_ nanoparticles or milk proteins, all concentrations of TiO_2_ nanoparticles and BPA were chosen to fall within the linear dynamic range (from the limit of quantitation to the upper limit of linearity) for each compound to generate a good signal-to-noise ratio. Specifically, the concentration of TiO_2_ nanoparticles could not be so high as to clog the ESI intake capillary, the concentration of BPA could not exceed its solubility in water, and the concentration of sodium formate could not go beyond its maximum effectiveness level. By direct infusion and using positive polarity, a typical full-scan ESI-ITMS spectrum of Millipore water exhibited, in Figure 1a, a peak at *m*/*z* 250.95(±0.01) that was attributed to [BPA + Na]^+^ residue deposited inside the ESI source. The spectrum of BPA (100 μg/mL) exhibited the *m*/*z* 250.95 peak and an intense peak at *m*/*z* 247.96(±0.01) of unknown structure assignment (tentatively, [C_9_H_11_O + CF_3_COOH]^+^) in Figure 1b [71]. In addition, new peaks appeared at *m*/*z* 361.89, 475.28, 589.36, 703.11, and 816.76. A search of the scientific literature suggested that the regular spacing (∆*m*/*z*) or repeating unit of 114 might be due to trifluoroacetic acid (TFA) contamination [72]. These unknown peaks that seemed to arise from TFA adduction on the fragment could be subject to further investigation by MS/MS, if deemed necessary. Although protein-TFA adducts are well known [73], it was surprising for several TFA molecules to associate with a single BPA molecule. A standard calibration curve was constructed for the ESI-ITMS determination of bisphenol A in water, using a setting of *m*/*z* 251 for extracted ion monitoring in positive polarity. Data points were obtained with an accumulation time of 0.3 ms for twenty different concentrations that covered nearly three orders of magnitude, going below the 1 μg/mL concentration level, as shown in Figure S1. The limit of detection was 0.24 μg/mL, and the limit of quantitation was 0.80 μg/mL. Higher sensitivity could be attained by using a longer accumulation time (5–10 ms) to produce measurable peak intensities at even lower concentrations. This standard calibration looked good in terms of linearity and sensitivity, even though the vast majority of BPA determinations had previously been conducted by negative ESI-MS using *m*/*z* 227 in MS mode or the fragment ions 133 and 147 in MS/MS mode. No attempt was made to quantitatively compare the sensitivity obtained in positive ESI (using sodium adduct) with routine LC-negative ESI-MS/MS due to our observation of interference by TiO_2_ peaks in the negative ESI mass spectrum. For real-world samples, the MS/MS function (which is normally with higher specificity and sensitivity) could be activated to eliminate or reduce all possible interferences in both qualitative and quantitative analyses.

### 2.2. Bisphenol A Adsorption onto Titanium Dioxide Nanoparticles

**Figure 2:**
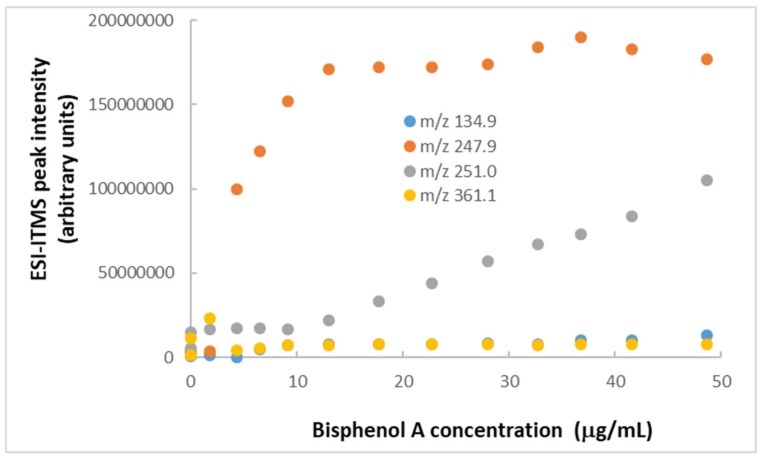
Standard calibration curves from serial dilutions of BPA (100 μg/mL) in mixtures with TiO_2_ nanoparticles (414 μg/mL) by ESI-ITMS, using extracted ion monitoring of four peaks at different *m*/*z* in positive polarity. Relative standard deviation of each data point = 5%.

Based on their exceptional physicochemical properties, TiO_2_ nanoparticles are very likely to adsorb organic contaminants in water [74]. In our study, BPA was chosen as a representative endocrine-disrupting compound to model the adsorption of emerging organic contaminants in water onto colloidal TiO_2_ nanoparticles. The hydroxyl functional groups and surface charge on the nanoparticles could be the main promoter of BPA adsorption via hydrogen-bonding and ion-π interaction. To determine if there were changes of BPA concentration after mixing with TiO_2_ nanoparticles, BPA standard solutions (100 μg/mL = 0.44 mM) were spiked with TiO_2_ nanopowder to attain different concentrations (from 20 μg/mL up to 144 μg/mL). After adding TiO_2_ nanopowder (128 μg/mL) to the BPA solution, no significant changes in ESI-ITMS peaks were observed, except for the reappearance of *m*/*z* 250.97 for [BPA + Na]^+^. Upon addition of 414 μg/mL TiO_2_ nanopowder to the BPA solution, the peak at *m*/*z* 250.96 diminished, while the peak at *m*/*z* 247.96 became dominant, as shown in Figure 1c. Yet, its intensity of 1.8 × 10^7^ arbitrary units was significantly lower than that of 4.0 × 10^7^ arbitrary units in Figure 1b, indicating a decrease of [BPA + Na]^+^ abundance due to approximately 55% adsorption of BPA on the TiO_2_ nanoparticles. Numerous low-intensity peaks appearing along the baseline from *m*/*z* 100 to *m*/*z* 1200 could be ascribed to a distribution of TiO_2_ nanoparticles with different sizes carrying various positive charges originating from TiO^+^ [75]. It should be noted that BPA contains a hydrogen atom at the tertiary carbon atom in the α-position of each benzene ring and a hydroxyl group [20], enabling mass spectrometric detection of the deprotonated molecular and product ions using negative polarity as well. Interestingly, the negative-polarity ESI-ITMS spectrum showed reproducible peaks at *m*/*z* 455.95–457.69 for [2BPA − H]^−^ (spectrum not shown), albeit at a lesser intensity (and hence, lower sensitivity for quantitative analysis) than those peaks observed above using positive polarity. ESI-ITMS was performed on a BPA standard solution (100 μg/mL) containing TiO_2_ nanopowder (414 μg/mL) using positive polarity. Standard calibration curves were constructed by serial dilution to measure the extracted ion counts for four peaks of different *m*/*z* values. As shown in Figure 2, *m*/*z* 251.0 is the best peak for quantitative analysis of BPA from 10 μg/mL up to 50 μg/mL. A higher sensitivity was attained for *m*/*z* 134.9 at BPA concentrations below 10 μg/mL, but fluctuations of ion distributions between *m*/*z* 251.0 and *m*/*z* 247.9 proved challenging. One plausible explanation was contamination by sodium, which is one of the most abundant contaminants in solvents; even HPLC grade solvents contain 0.1 μg/mL of sodium ions or more. Sodium contamination can also leach out of the glassware (used for sample preparation) over time.

### 2.3. Adduction and Desorption Effects of Sodium Formate

To alleviate the intensity fluctuations mentioned above, a small excess of sodium ions was added to the BPA standard solution in mixtures with TiO_2_ nanopowder. Using sodium formate, the *m*/*z* 247.9 peak was suppressed, while the *m*/*z* 251.0 peak was enhanced, to generate a very stable peak intensity, as shown in Figure 1d after the addition of sodium formate for comparison with Figure 1c before the addition of sodium formate. Their percentage changes are summarized in Table 1 to indicate particularly a significant increase of the peak intensity at *m*/*z* 251.0 at the expense of a decreased peak intensity at *m*/*z* 247.9. Apparently, sodium ion adduction, using either the formate or the acetate, produced the [BPA + Na]^+^ ion and suppressed the unknown ion. It was previously reported that the fragmentation pattern of the protonated adduct precursor ion drastically differed from the fragmentation pattern of the sodium adduct precursor ion [76]. This adduction effect was beneficial to the present work, since the peak intensity appeared to be very stable for the [BPA + Na]^+^ ion, for improved precision in the quantitative analysis.

**Figure 3:**
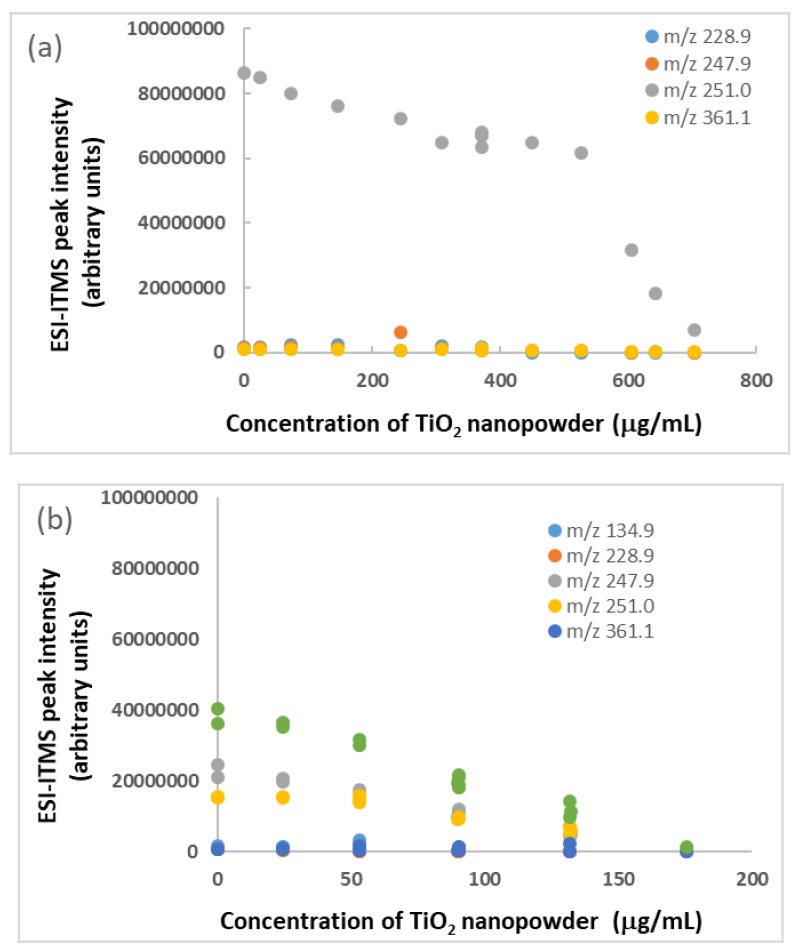
Binding of TiO_2_ nanoparticles with BPA in standard solution (1.8 μg/mL): (**a**) containing sodium formate (41 μg/mL) and (**b**) in absence of sodium formate. Relative standard deviation of each data point = 5%.

The effect of formate on BPA binding with TiO_2_ nanoparticles was studied further by adding TiO_2_ nanopowder to the BPA standard solution (1.8 μg/mL) containing sodium formate (41 μg/mL). As shown in Figure 3a, the *m*/*z* 251.0 peak intensity decreased gradually with the increasing addition of TiO_2_ nanopowder. Apparently, the higher peak intensities than those shown in Figure 3b suggest that formate competed against BPA for the binding sites on TiO_2_. It might also prevent the nanoparticles from early aggregation, as the ESI-ITMS instrument could tolerate the direct infusion of TiO_2_ nanopowder up to a concentration of 500 μg/mL after every solution was sonicated for 5 min before analysis. Upon addition of more TiO_2_, the nanopowder began to settle down on the flask bottom due to aggregation. The *m*/*z* 251.0 peak intensity decreased rapidly as well, most likely due to gradual clogging of the ESI stainless steel capillary tube that was used in sample injection. In other words, the peak intensity observed in ITMS depended on both the BPA concentration and the actual infusion rate (nominally at 10 μL/min). This explanation seemed reasonable, especially given that aggregation scaled with nanoparticle concentration. As the signal intensity could be lost due to both sedimentation and clogging, it stands to reason that after a certain concentration threshold, when nanoparticle aggregates were large enough to clog the capillary, signal intensity decreased at an increasing rate. In comparison, Figure 3b does not show how much BPA could adsorb on TiO_2_ nanoparticles in the absence of sodium formate due to a randomly varying distribution of ionized BPA between *m*/*z* 247.9 and *m*/*z* 251.0. This challenge was alleviated when these two peak intensities were summed, and their sum (green data points) decreased linearly with increasing TiO_2_ concentrations. By comparing the rate of peak intensity decrease, it was obvious that BPA did not bind efficiently with TiO_2_ nanoparticles in the presence of sodium formate, because the peak intensities of *m*/*z* 251.0 (corresponding to free BPA) in Figure 3a were significantly higher than those in Figure 3b. A previous work had determined a 40% efficiency for ammonium formate (2 mM) to desorb BPA from TiO_2_ nanoparticles [20]. Early aggregation of the nanoparticles (at concentrations below 175 μg/mL) was likely happening in the absence of formate, as evidenced by the straight decrease of peak intensity sum with increasing TiO_2_ concentrations (rather than following a curve in the shape of Langmuir isotherm). It seemed like a good practice to use sodium formate solution (41 μg/mL) to wash and, hence, clean the ESI intake capillary between consecutive samples during ITMS analysis.

**Figure 4:**
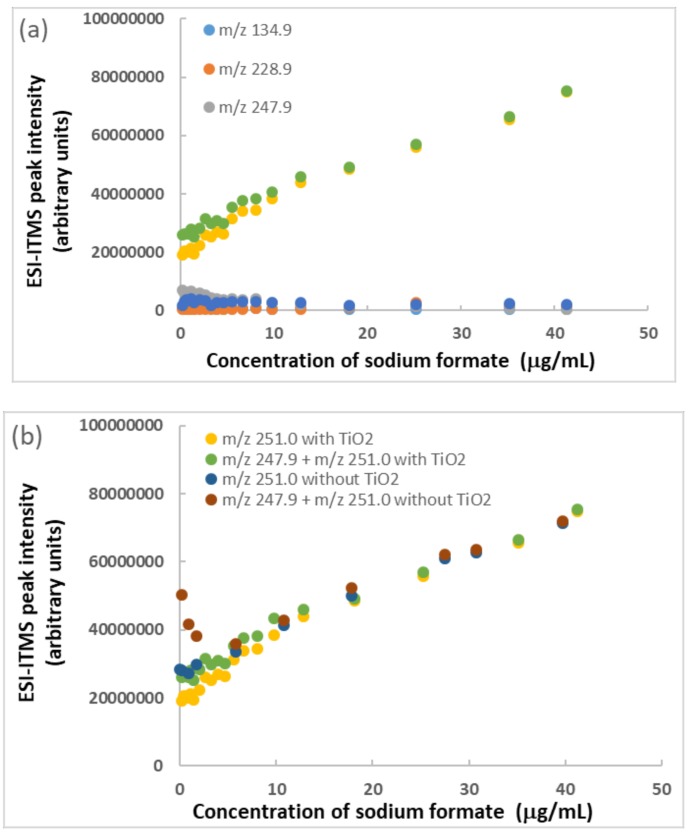
Effect of sodium formate concentration on minimization of *m*/*z* 247.9 peak intensity and maximization of *m*/*z* 251.0 peak intensity in ESI-ITMS analysis: (**a**) BPA standard solution (1.8 μg/mL) containing TiO_2_ nanoparticles (176 μg/mL) and (**b**) BPA standard solution (1.8 μg/mL) only for comparison. Relative standard deviation of each data point = 5%.

A systematic experiment was conducted to investigate how good sodium formate could be for desorption of BPA bound on TiO_2_ nanoparticles. ESI-ITMS analysis of bisphenol A (1.8 μg/mL) containing TiO_2_ nanoparticles (176 μg/mL) was completed using sodium formate concentrations as high as 41 μg/mL. After, the blank intensity due to sodium formate was subtracted from all data points before plotting. The peak intensity of *m*/*z* 251 for [M + Na]^+^ presented good results in Figure 4a, showing initially there was a slightly steeper slope along the yellow data points at low formate concentrations. This result suggested a progressive desorption of BPA from the TiO_2_ nanoparticles by the formate anion, on the assumption that adsorbed BPA was not detectable by ITMS. A minimum concentration of 18 μg/mL (= 0.26 mM) sodium formate was required for the sodium cation to minimize the intensity of *m*/*z* 247.9 peak and to maximize the intensity of *m*/*z* 251.0 peak. Above 18 μg/mL, the green data points (sum of *m*/*z* 247.9 and *m*/*z* 251.0) became the same as the yellow data points (*m*/*z* 251.0 only). For the confirmation of desorption, it became necessary to do a comparison between BPA plus sodium formate and BPA containing TiO_2_ nanoparticles plus sodium formate. As the positive slopes in Figure 4b indicate, the formate seemed capable of enhancing the ESI performance and, thus, increasing the peak intensities. It probably enhanced the ionization, such as when using formic acid. A literature search has revealed a previous report that sodium formate (0.001 mM) and formic acid (15 mM) were added to the LC mobile phase as electrolytes for ESI-positive mode in the MS analysis of glycerolipids [77]. Inclusion of ammonium formate (0.2 mM) generated a beneficial effect of increased ESI efficiency and capacity for LC-MS/MS analysis of flavonoids and ginkgolides in serum analysis [78]. Addition of ammonium formate (1 mM) increased the ionization efficiency of endocrine-disrupting chemicals by countering matrix effects in an environmental water analysis [79].

Optimization of the drying gas temperature in the ESI-ITMS analysis was deemed crucial at this stage of research. It was performed using 428 μg/mL TiO_2_ nanopowder in 1.8 μg/mL BPA standard solution containing 41 μg/mL sodium formate. As shown in Figure S2, 180 °C was good by producing a stable peak intensity on the plateau for *m*/*z* 251.0. Temperatures higher than 200 °C caused a significant decrease in the intensity, probably due to precipitation of sodium formate salt with TiO_2_ nanoparticles during ESI.

### 2.4. Impact of Amino Acids on TiO_2_ Nanoparticles

**Figure 5:**
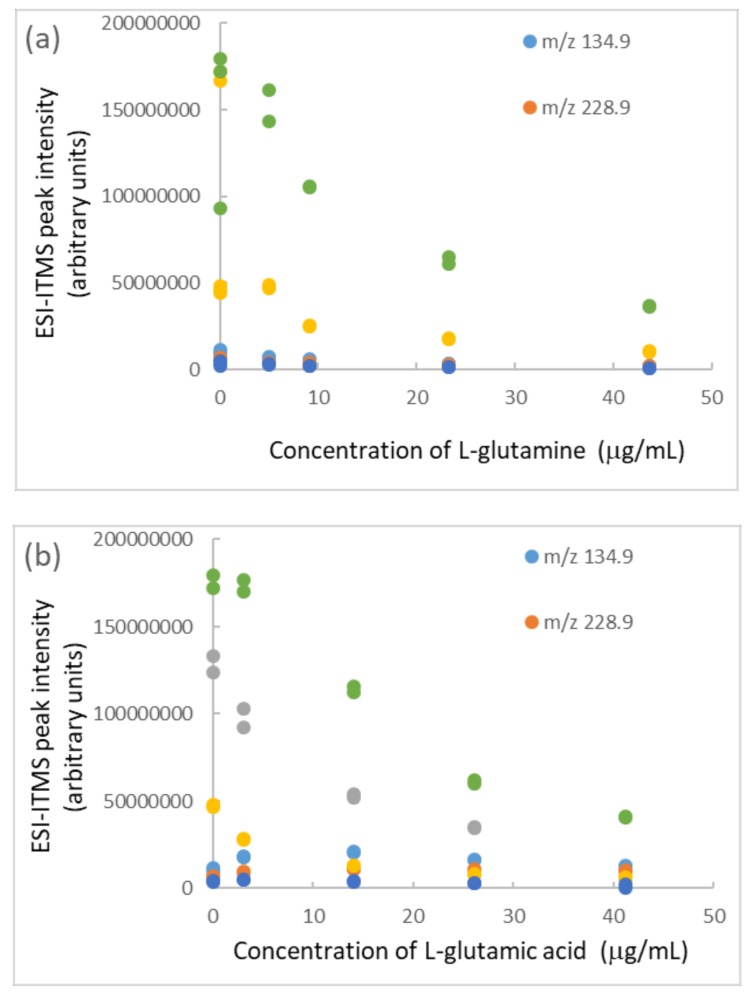
Impact of (**a**) l-glutamine and (**b**) l-glutamic acid on the ESI-ITMS analysis of BPA solution (25 μg/mL) containing TiO_2_ nanoparticles (104 μg/mL). Relative standard deviation of each data point = 5%.

Amino acids are essential nutrients in health foods and dietary supplements that occur either in the free forms or as proteins. They can be analyzed by single-quadrupole mass spectrometry with high throughput [80]. A combined mass spectrometry–nuclear magnetic resonance approach is efficient for their qualitative and quantitative analysis, with tolerances of ±10%–20%, which correspond to European recommendations [81]. Their inherent tendency to act as an eco-friendly capping agent during the synthesis of nanoparticles has been studied [82]. Adsorption of amino acids via the backbone on the negatively charged surface of amorphous TiO_2_ nanoparticles always happens [83]. Glutamic acid and glutamine in human milk increase 2.5 and 20 times with progressing lactation, representing more than 50% of total free amino acids at three months [84]. Cow’s milk contains 30 mg/L glutamic acid [85]. l-glutamic acid (l-Glu) exhibits an intrinsic peroxidase-like activity [86]; glutamate plays a vital role in many physiological processes and is involved in various neurological and psychiatric disorders [87]. The interaction of amino acids with the TiO_2_ (101) anatase surface has recently been investigated by means of periodic simulations, from both static and dynamic points of view. The adsorption energies of the complexes (ΔE^C^_ADS_) calculated for l-Glu and l-glutamine (l-Gln) were as high as −112.6 and −127.5 kJ/mol, respectively [88]. In the present study, these two amino acids were assessed for their impact on the ESI-ITMS analysis of BPA (25 μg/mL) containing TiO_2_ nanoparticles (104 μg/mL). Both amino acids caused sedimentation of the nanoparticles in the BPA solution, as shown by a decrease of peak intensities with increasing amino acid concentrations (beyond their natural concentrations in cow milk) in Figure 5. TiO_2_ nanoparticles and amino acids carried charges, depending on sample pH versus their point of zero charge and pK_a_ values. If they neutralized each other, precipitation could occur. Indeed, precipitation can remove adsorbed BPA and result in reduced intensities. Fortunately, after sonicating the ESI intake capillary in water for 5 min, the *m*/*z* 247.9 peak intensity restored to 1.42 × 10^8^ (as compared with 1.76 × 10^8^ observed before any amino acid additions).

**Figure 6:**
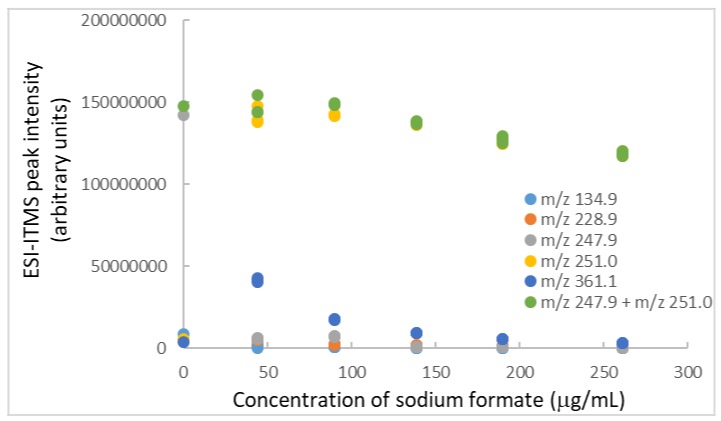
Impact of sodium formate on ESI-ITMS analysis of BPA solution (25 μg/mL) containing TiO_2_ nanoparticles (104 μg/mL) and l-glutamic acid (41 μg/mL). Relative standard deviation of each data point = 5%.

When sodium formate was added to the last BPA solution containing TiO_2_ nanoparticles and l-glutamic acid, it worked to resuspend the sedimented TiO_2_ nanoparticles after ultrasonication for only 3 min. The BPA peaks returned to their original intensities, but they decreased gradually with increasing sodium formate concentrations, as shown in Figure 6. This decrease might mean sodium formate could not fully prevent sedimentation of the nanoparticles. After sonicating the ESI intake capillary to clean out any sediment, the *m*/*z* 247.9 + *m*/*z* 251.0 peak intensity (1.25 × 10^8^) was nearly the same as that (1.19 × 10^8^) obtained before sonication (thus, indicating no sedimentation of nanoparticles inside the capillary). One interpretation was that too high a concentration of sodium formate (above 90 μg/mL or 1.3 mM) decreased the ESI efficiency (by approximately 20% at 260 μg/mL), even though it successfully prevented the sedimentation (that tended to clog the intake capillary).

The analytical merits of sodium formate were further explored by standard additions with BPA solid to spike the above BPA solution (25 μg/mL) containing TiO_2_ nanoparticles (104 μg/mL), l-glutamic acid (41 μg/mL), and sodium formate (260 μg/mL or 2.4 mM). After the first addition, followed by 5 min of ultrasonication, the BPA peaks (at *m*/*z* 249.7 and *m*/*z* 251.0) did not increase their intensities significantly, as shown in Figure S3 (from the leftmost data point to the next data point). When the second addition was followed by 10 min of ultrasonication (and 10 min of quiescence to cool the sample solution down to room temperature), the BPA peak intensity at *m*/*z* 251.0 increased in proportion to the amount of BPA added. This linear trend continued up to a BPA concentration of 160 μg/mL (or 0.70 mM), at least, with a slope of y = 2.0 × 10^6^ similar to that displayed above, in Figure S1 for BPA concentrations below 65 μg/mL. It was notable that sodium formate helped to dissolve solid BPA without needing to use methanol, presumably due to the association of BPA with formate to form an adduct similar in chemical structure to BPA formaldehyde [89]. No clogging of the ESI capillary occurred throughout the many consecutive ITMS analyses over several hours. Empirically speaking, sodium formate seemed to be a chemical agent that ameliorated some practical challenges in BPA analysis. It facilitated the sodium adduction of BPA, producing a strong peak at *m*/*z* 251.0 for [BPA − Na]^+^ that was approximately 400 times more intense than the weak peak at *m*/*z* 249.7.

### 2.5. BPA in Milk Analysis

Containing sodium formate in the 1.8 μg/mL standard solution, BPA was determined by ESI-ITMS before and after cow milk was added in different volumes—the initial and final mass spectra are presented in Figure S4a,b. As shown in Figure S5, milk proteins apparently adsorbed BPA to decrease the peak intensity of *m*/*z* 251.0 for [BPA + Na]^+^. All the peak intensities were already corrected for dilution of the BPA standard solution by milk based on the assumption that signals will decrease linearly with dilution in the concentration dynamic range. Another plausible explanation was that direct injection of milk without pretreatment (even after dilution, particularly for the larger volumes) might cause contamination of the spectrometer parts, which could decrease the ionization efficiency as well. However, low ionization efficiency could be ruled out merely by the experimental design that the original BPA solution was analyzed after the milk additions (from 20 μL to 140 μL), followed by analyses of a couple small addition volumes (5 μL and 10 μL). As no alternative explanations could be found, BPA adsorption onto milk proteins continued to be the prime suspect. It had previously been reported that BPA had a high tendency to interact with the fatty compounds in milk [90]. A previous study on the binding capacity of bisphenol A with erythrocyte proteins (hemoglobin, catalase, and glutathione peroxidase) has shown the presence of various hydrogen bonds of BPA with the proteins [91]. All these results and ours support a recent study that reported the content of free BPA was higher in infant formulas than human milk [92].

In order to test the above suspicion, hydrochloric acid (2 N) was added to curdle the milk, and the casein protein curds were filtered off. This curdling dramatically reduced the protein concentration in the milk by approximately 80% [93]. The whey was then used to repeat the BPA standard solution analysis by ESI-ITMS. As presented in Figure S4c, significantly less BPA was being adsorbed, and the decrease of [BPA + Na]^+^ peak intensity became less (than that observed after milk addition). As shown in Figure S6, which plots the normalized peak intensity versus the volume of milk/whey added to the BPA standard solution, the difference between the two curves can be attributed to the removal of milk proteins by curdling. The whey curve suggests that some matrix effect remained, probably due to the adsorption of BPA by the remaining 20% of milk proteins. Else, a higher viscosity of whey than water hampered the ESI performance. The protein in cow’s milk is 20% whey protein and 80% casein protein. The protein fraction in whey constitutes approximately 10% of the total dry solids in whey. It consists of approximately 50% ß-lactoglobulin, 20% α-lactalbumin, blood serum albumin, immunoglobulins, lactoferrin, and transferrin, plus many minor proteins, including enzymes [94]. Future work could add 0.8% chitosan to coacervate the milk proteins over an incubation period of one hour. Approximately 86% of the milk proteins would be present in the pellet fraction, and the protein concentration in the supernatant fraction would decrease from 29.4 to 4.2 mg/mL [95].

The next investigation aimed at BPA binding with whey proteins, lipids, and lactose. The addition of whey (0.5 mL) to 1.8 mg/mL BPA standard solution (25 mL) initially decreased the ESI-ITMS intensity at *m*/*z* 250.1 for [BPA + Na]^+^ by approximately 50% (from 8.25 × 10^7^ before to 4.39 × 10^7^ after whey addition), as shown by the two leftmost data points at 0 μg/mL TiO_2_ nanopowder in Figure S7. Then, TiO_2_ nanopowder was added intentionally to adsorb free BPA molecules in the solution containing whey. It was surprising to observe no significant decrease in the *m*/*z* 251.0 peak intensity even after several additions, up to a final concentration of 428 μg/mL TiO_2_ nanopowder. This observation was in stark contrast with the results presented above in Figure S5. There were apparently no free BPA molecules available for adsorption by the nanoparticles, which implied that all the BPA probably bound with the whey proteins, lipids, and lactose. This also suggested that ESI could release the bound BPA from the whey proteins to form [BPA + Na]^+^ adduct ions for quantitative analysis by ITMS. In other words, BPA whey proteins denatured at ESI because the source temperature was sufficiently high, but BPA lipids/lactose persisted through ESI. This could explain why having whey proteins/lipids/lactose gave a lower signal than BPA alone. Although milk proteins might denature at ESI too, they are more heat-stable than whey [96]. Hence, BPA binding with whey proteins, lipids, lactose, and butter (80%–82% milk fat, 16%–17% water, and 1%–2% protein) could deem crucial for future investigation. A dilution of 4%-fat milk (7.5 g) with Millipore water to a final volume (20 mL) containing 375 μg/mL sodium formate exhibited only a low peak intensity (9.4 × 10^5^) at *m*/*z* 251.0 even after the addition of BPA (327 μg/mL). Intuitively, the BPA either dissolved improperly or strongly bound with the milk proteins. A previously presented precipitation process has combined EDTA-McIlvaine buffer (the common precipitation agent) with acetonitrile to facilitate the transference of BPA to the supernatant [84]. When tested in our lab, mixing acetonitrile (3 mL) with the above diluted milk solution (19 mL) exhibited a slightly increased peak intensity (3.7 × 10^6^) at *m*/*z* 251.0 that was, however, significantly lower than expected (4.0 × 10^8^ for 178 μg/mL BPA).

## 3. Materials and Methods

### 3.1. Materials

All chemicals were obtained from commercial sources. Bisphenol A (C_15_H_16_O_2_, M.W. = 228.11503 g/mol); l-glutamic acid (C_5_H_9_NO_4_, M.W. = 147.13 g/mol); l-glutamine (C_5_H_10_N_2_O_3_, M.W. = 146.14 g/mol); methanol (LC/MS grade); sodium formate (HCOONa, M.W. = 68.01 g/mol); and TiO_2_ nanoparticles (99% anatase) were purchased from Sigma-Aldrich (Darmstadt, Germany). Acetonitrile (GC grade) was obtained from Fluka (Buchs, Switzerland). Ultrapure water, from a Milli-Q system (Millipore, Milford, MA, USA), was used to prepare all analyte and desorption solutions.

### 3.2. Methods

#### 3.2.1. Adsorption of BPA on TiO_2_ Nanoparticles

Bisphenol A is a solid with water solubility of 120–300 milligrams per liter (and a greater solubility at alkaline pH values) [97]. A weighed quantity of TiO_2_ nanopowder was added to 100 mL BPA solution, yielding a concentration of 20–144 μg/mL. The mixture was homogenized by sonication for 5–30 min before ESI-ITMS analysis to determine the initial BPA concentration. After being kept in the dark to achieve adsorption-desorption equilibrium overnight, the mixture was analyzed by ESI-ITMS again to determine the final BPA determination. A *p*-value <0.05 was considered statistically significant in the validation of BPA adsorption onto TiO_2_ nanoparticles based on the difference between the initial and final concentrations.

#### 3.2.2. Desorption of BPA from TiO_2_ Nanoparticles

To investigate whether sodium formate was efficient for the desorption of BPA bound on TiO_2_ nanoparticles, a standard solution of BPA (1.8 μg/mL) containing TiO_2_ nanoparticles (176 μg/mL) was spiked with sodium formate at concentrations up to 41 μg/mL for ESI-ITMS analysis. The blank intensity due to sodium formate contaminants was subtracted from all data points before plotting.

#### 3.2.3. Milk Curdling and Filtration

Hydrochloric acid (2 M) (TitriPUR grade, Merck; Darmstadt, Germany) was added dropwise to cow milk (5.0 g, Die Ergiebige 10% fett or Die Leichte 4% fett, Bären Marke; Germany) until curdling occurred. The curdled milk was poured and washed into a glass funnel for straining through Whatman no. 1 filter paper. The filtrate, also known as strained whey, was neutralized with sodium hydroxide solution (1 N) (Titrisol grade, Merck; Darmstadt, Germany) to pH 7.00 ± 0.05, as indicated by a pH meter (calibrated against 3.0 M KCl solution). The neutral whey was added in small aliquots to a BPA standard solution for ESI-ITMS analysis after complete mixing. A comparative experiment was also conducted by adding cow milk directly to a similar BPA standard solution.

### 3.3. Instrumentation

Full-scan mass spectra were acquired by using a Bruker AmaZon Speed ion trap instrument equipped with an ESI-source (Bruker Daltonik GmbH; Bremen, Germany) operated in the positive ionization mode. The MS was operated in a scanning mode at 32,500 *m*/*z* per second. Each mass spectrum represented the average of 50 scans that were performed by direct infusion of analyte solutions of pure compounds in methanol/water containing sodium formate (typically 41 μg/mL) with a flow rate of 10 μL/min, aided by pressurized nitrogen as a nebulizing gas [98]. Additionally, a heated drying gas flowed against the ions assisting desolvation and the removal of neutrals (180 °C, 4.0 L/min). The spray assembly was held at ground potential, and ions were focused onto the entrance of a metal-coated glass capillary held 4500 V below the sprayer, with an end plate offset of 500 V. The exit side of the capillary was also metal-coated and carried a potential of 100 V. During the accumulation time (typically 0.3 ms), analyte ions entered the trap through the first end-cap electrode, where they were trapped by a radio frequency field of 400 V_pp_ at 781 kHz. Detection of ions by a conversion dynode was performed using the extracted ion current. Each spectrum was obtained by averaging 5 sequential scans, with a rolling averaging number of 50. Complete settings for the ESI-ITMS operation are summarized in Supplementary Table S1. Data acquisition was conducted by TrapControl software (version 7.0, Bruker; Bremen, Germany); experiments were performed by scanning from *m*/*z* 70 to *m*/*z* 2000 with enhanced resolution. Bisphenol A was identified by correlating its sodium adduct ion (*m*/*z* 251.0), main fragment ion (*m*/*z* 134.9), and unassigned product ion (*m*/*z* 247.9) with both a commercial standard and literature data. At least three replicates were run for the data shown in all the figures presented above.

## 4. Conclusions

Sodium adduction, using either sodium formate or sodium acetate, produced the [BPA + Na]^+^ ion at *m*/*z* 251.0 and suppressed the unknown ion at *m*/*z* 247.9. These chemical agents were deemed capable of enhancing the ESI efficiency and, thus, increasing the peak intensities. Specifically, 260 μM sodium formate was required to minimize the intensity of *m*/*z* 247.9 peak and to maximize the intensity of *m*/*z* 251.0 peak for 7.9 μM bisphenol A—their mole ratio was approximately 32:1. Furthermore, sodium formate prevented TiO_2_ nanoparticles from aggregation and sedimentation so that the ESI-ITMS instrument could operate properly during their direct infusion up to 500 μg/mL without clogging the intake capillary. Even if l-glutamic acid or l-glutamine caused sedimentation of TiO_2_ nanoparticles in a BPA solution, the addition of sodium formate (1.3 mM) resuspended the TiO_2_ nanoparticles after ultrasonication for only 3 min. In milk analysis, proteins significantly adsorbed BPA to decrease the [BPA + Na]^+^ peak intensity down to 9%; removal of milk proteins by curdling brought the intensity back to 41%. Evidently, milk whey allowed desorption of bound BPA during ESI for ITMS analysis. Additions up to 428 μg/mL of TiO_2_ nanopowder confirmed the absence of free BPA in the milk whey. These findings provide a deeper insight than into the unique mechanism of adsorption and desorption interactions that could otherwise compromise the accuracy of, or certainty and confidence in, BPA quantitative analysis by ESI-ITMS. The present study could be extended to further optimize all ESI-ITMS operational conditions for accurate determinations of BPA in food, personal care, and pharmaceutical products containing dairy protein and other oxide nanomaterials. Besides ITMS, many analytical methods will be used to help disclose the details of the formed compound at *m*/*z* 247.9 in our future work.

## Figures and Tables

**Table 1:** Effects of sodium formate and acetate on electrospray ionization-ion trap mass spectronomy (ESI-ITMS) peak intensities (and their percentage changes) for bisphenol A (BPA) at *m*/*z* 134.9, *m*/*z* 247.9, and *m*/*z* 251.0.

BPA Concentration (μg/mL)	Sodium Formate Concentration (μg/mL)	Sodium Acetate Concentration (μg/mL)	*m*/*z* 134.9	*m*/*z* 179.9 *	*m*/*z* 247.9	*m*/*z* 251.0	*m*/*z* 361.1 *
1.8	0	0	3.20 × 10^6^	1.80 × 10^5^	1.64 × 10^7^	2.32 × 10^7^	1.80 × 10^6^
1.8	0	50	1.40 × 10^5^	1.70 × 10^5^	5.00 × 10^5^	7.80 × 10^7^	7.30 × 10^5^
			−96%	−6%	−97%	236%	−59%
1.8	41	0	2.00 × 10^5^	7.00 × 10^4^	9.90 × 10^5^	7.80 × 10^7^	1.05 × 10^6^
			−94%	−61%	−94%	236%	−42%

* Peak intensities for impurities at *m*/*z* 179.9 and *m*/*z* 361.1 are included as negative controls for comparison only.

## Supplementary Materials

The following are available online at https://www.mdpi.com/1420-3049/25/3/708/s1: Figure S1: Standard calibration curve for determination of bisphenol A by ESI-ITMS, using *m*/*z* 251.0 for extracted ion monitoring in positive polarity. Figure S2: Optimization of drying gas temperature for TiO_2_ nanopowder (428 μg/mL) in BPA standard solution (1.8 μg/mL) containing sodium formate (41 μg/mL). Figure S3: Addition of BPA into BPA solution (25 μg/mL) containing TiO_2_ nanoparticles (104 μg/mL), l-glutamic acid (41 μg/mL), and sodium formate (260 μg/mL) for ESI-ITMS analysis. Figure S4: ESI-ITMS spectra: (a) 1.00 mL of BPA (1.8 μg/mL) + 50 μg/mL sodium formate, (b) 0.14 mL of milk + 1.00 mL of BPA (1.8 μg/mL) + 50 μg/mL sodium formate, and (c) 0.14 mL of whey + 1.00 mL of BPA (1.8 μg/mL) + 50 μg/mL sodium formate. Figure S5: ESI-ITMS analysis of BPA (1.8 μg/mL) standard solution containing sodium formate (41 μg/mL) after adding different volumes of milk. Figure S6: ESI-ITMS analysis of BPA (1.8 μg/mL) standard solution containing sodium formate (41 μg/mL) after adding different volumes of milk or whey. All peak intensities were normalized for easy comparison between milk and whey. Figure S7: Effect of TiO_2_ nanoparticles on BPA binding with whey proteins. Addition of whey (0.5 mL) to 1.8 mg/mL BPA standard solution (25 mL) initially at 0 μg/mL TiO_2_ decreased the ESI-ITMS intensity of *m*/*z* 250.1 for [BPA + Na]^+^ by approximately half. Table S1: Operational settings for ESI-ITMS analysis.
