# Supplementary material for: Ion-Trap Mass Spectrometric Analysis of Bisphenol A Interactions With Titanium Dioxide Nanoparticles and Milk Proteins
Source: Molecules. 2020 Feb 6;25(3):708. doi: 10.3390/molecules25030708 (PMC7037553; doi:10.3390/molecules25030708)
Supplement: Supplementary file 1 [file molecules-25-00708-s001.pdf]

Article

Ion-trap Mass Spectrometric Analysis of Bisphenol A Interactions with Titanium Dioxide Nanoparticles and Milk Proteins

Edward P.C. Lai [1,*], Hendrik Kersten [2] and Thorsten Benter[2]

[1] Ottawa-Carleton Chemistry Institute, Department of Chemistry, Carleton University, Ottawa, ON K1S 5B6, Canada; edward.lai@carleton.ca

[2] Institute for Pure and Applied Mass Spectrometry, Physical and Theoretical Chemistry, Bergische Universität Wuppertal, Gaussstr.20, 42119 Wuppertal, Germany; hkersten@uni-wuppertal.de, tbenter@uni-wuppertal.de

* Correspondence: edward.lai@carleton.ca; Tel.: +01-613-520-2600

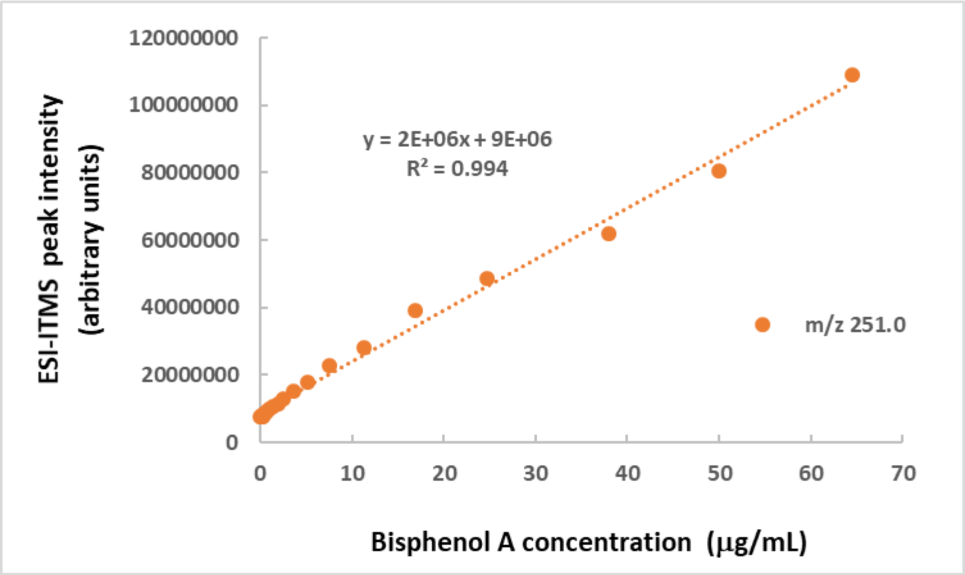

Figure S1. Standard calibration curve for determination of bisphenol A by ESI-ITMS, using m/z 251.0 for extracted ion monitoring in positive polarity. Relative standard deviation of each data point = 5%.

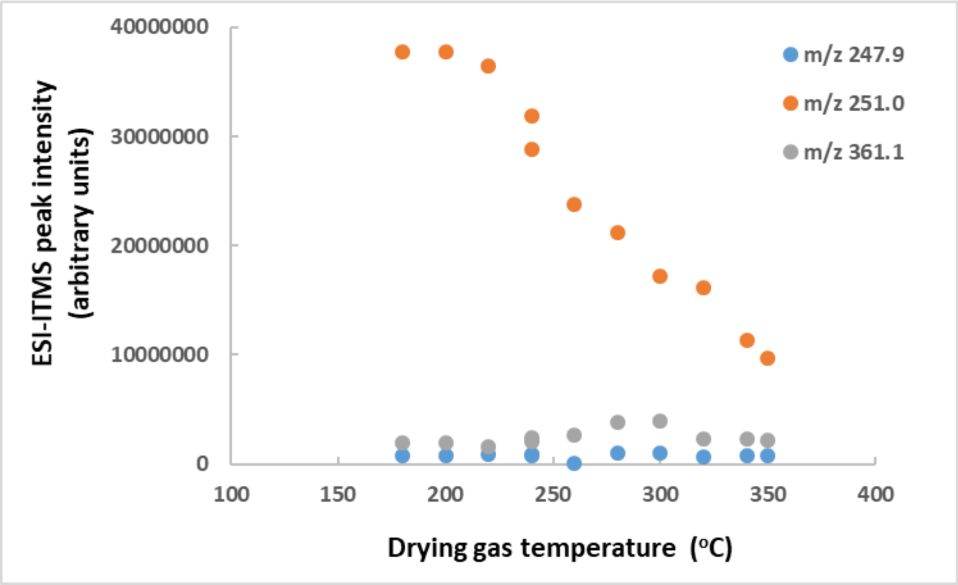

Figure S2. Optimization of drying gas temperature for TiO_2 nanopowder (428 g/mL) in BPA standard solution (1.8 g/mL) containing sodium formate (41 g/mL). Relative standard deviation of each data point = 5%.

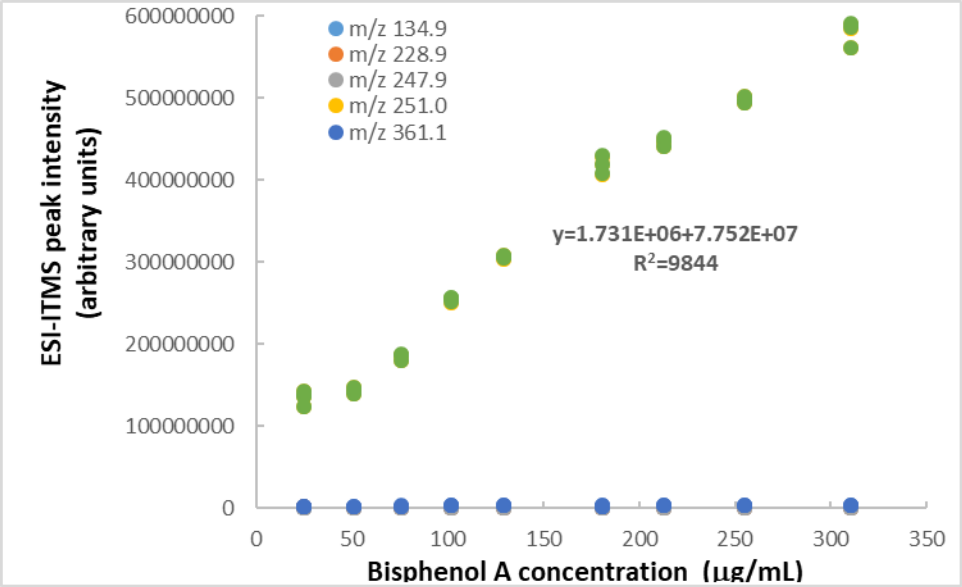

Figure S3. Addition of BPA into BPA solution (25 g/mL) containing TiO_2 nanoparticles (104 g/mL), L-glutamic acid (41 g/mL) and sodium formate (260 g/mL) for ESI-ITMS analysis. Relative standard deviation of each data point = 5%.

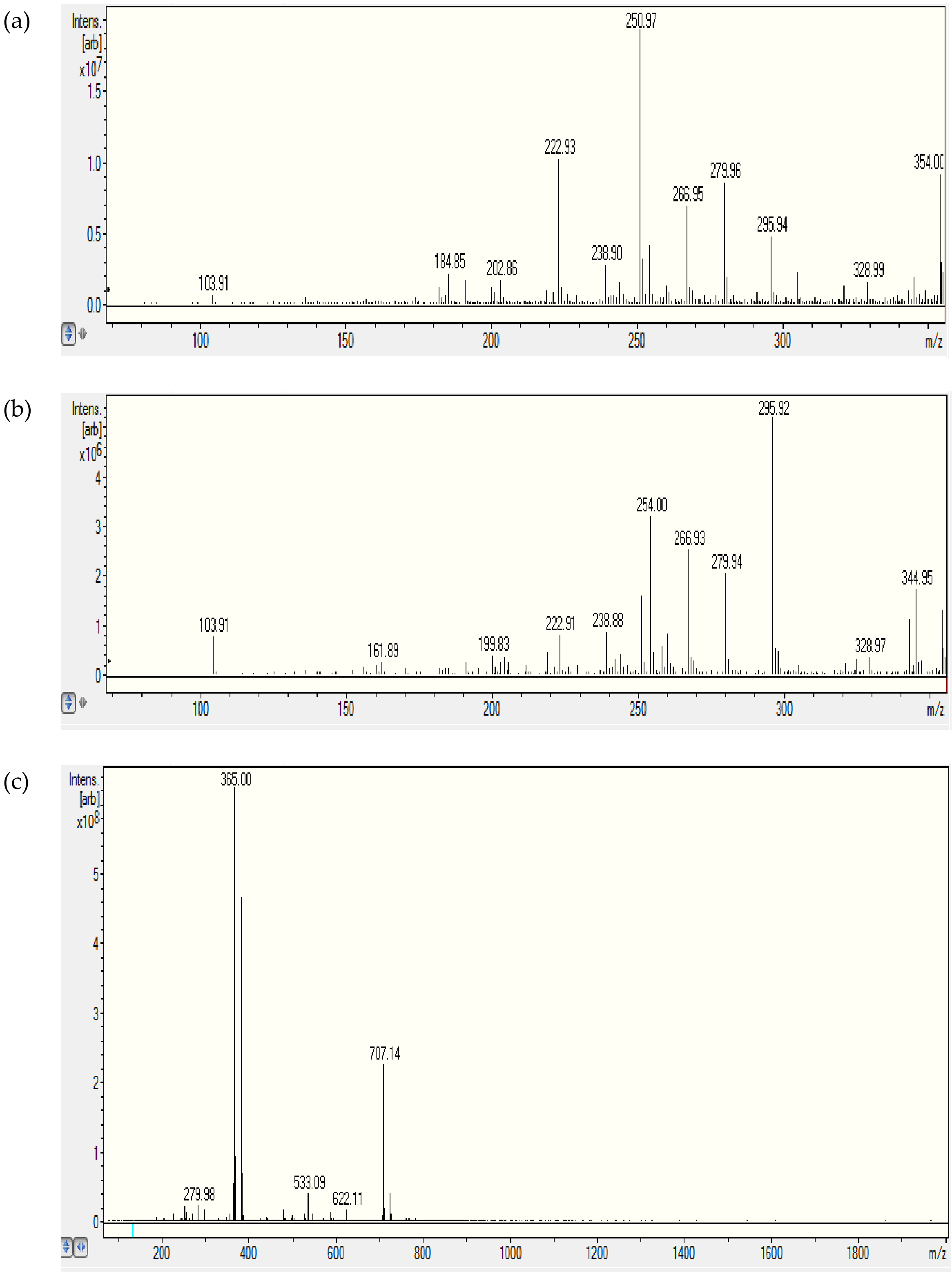

Figure S4. ESI-ITMS spectra: (a) 1.00 mL of BPA (1.8 g/mL) + 50 g/mL sodium formate, (b) 0.14 mL of milk + 1.00 mL of BPA (1.8 g/mL) + 50 g/mL sodium formate, (c) 0.14 mL of whey + 1.00 mL of BPA (1.8 g/mL) + 50 g/mL sodium formate.

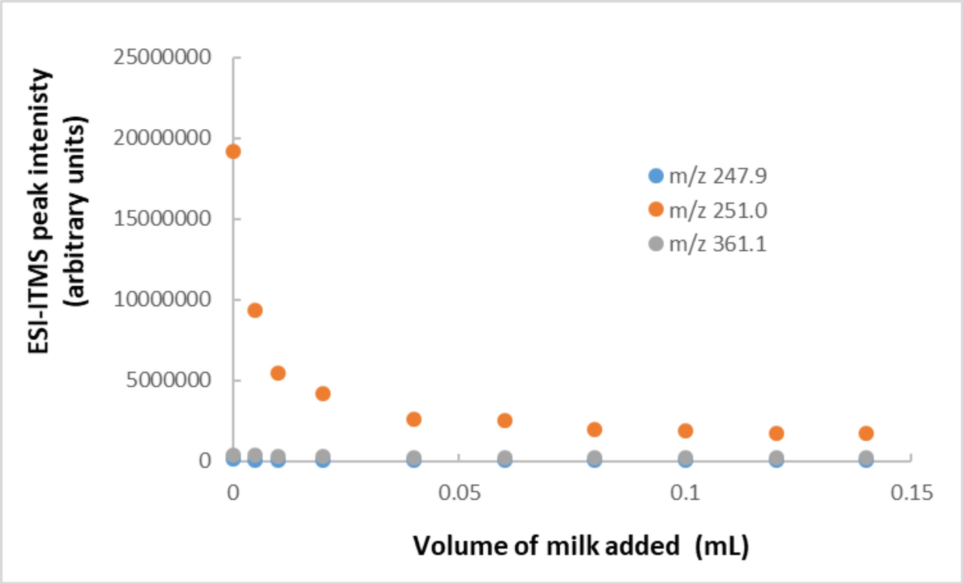

Figure S5. ESI-ITMS analysis of BPA (1.8 g/mL) standard solution containing sodium formate (41 g/mL) after adding different volumes of milk. Relative standard deviation of each data point = 5%.

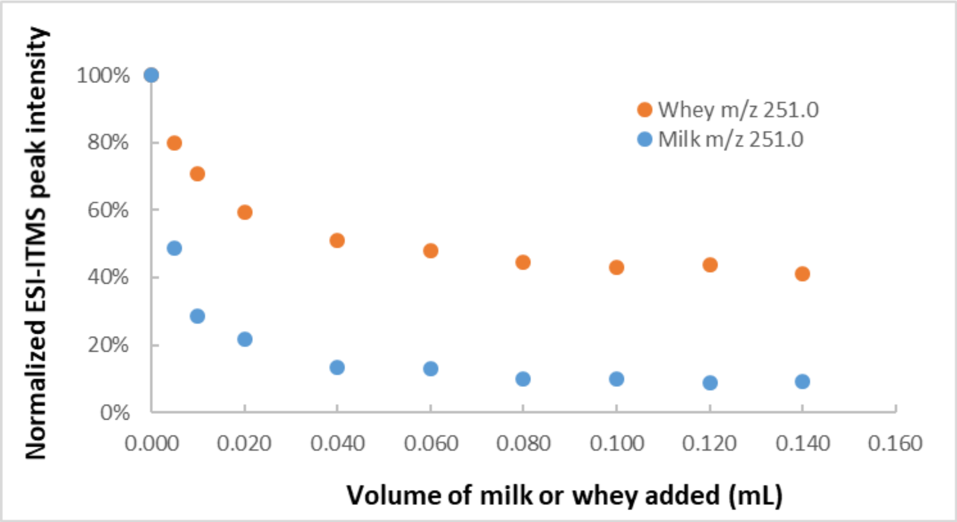

Figure S6. ESI-ITMS analysis of BPA (1.8 g/mL) standard solution containing sodium formate (41 g/mL) after adding different volumes of milk or whey. All peak intensities were normalized for easy comparison between milk and whey. Relative standard deviation of each data point = 5%.

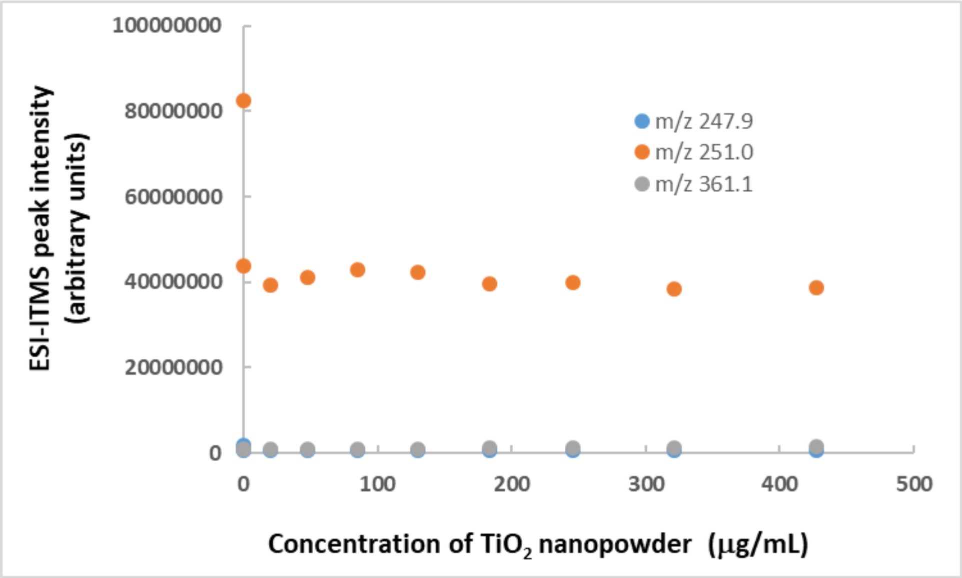

Figure S7. Effect of TiO_2 nanoparticles on BPA binding with whey proteins. Addition of whey (0.5 mL) to 1.8 mg/mL BPA standard solution (25 mL) initially at 0 g/mL TiO_2 decreased the ESI-ITMS intensity of m/z 250.1 for $[BPA+Na]^+$ by approximately half. Relative standard deviation of each data point = 5%.

Table 1. Operational settings for ESI-ITMS analysis.

Mass Range Mode	UltraScan
Ion Polarity	positive
Mass scan range	70-2000 Da
Ion Source Type	ESI
Alternating Ion Polarity	off
Source (capillary/interface) temperature	250°C
Capillary (spray voltage)	4500 V
End plate offset	50 V
Nebulizer gas	N_2 99.9990% (0.5 bar)
Dry gas	N_2 99.9990% (4.0 L/min)
Dry gas temperature	180°C
